# Longer Leukocyte Telomeres Are Associated with Ultra-Endurance Exercise Independent of Cardiovascular Risk Factors

**DOI:** 10.1371/journal.pone.0069377

**Published:** 2013-07-31

**Authors:** Joshua Denham, Christopher P. Nelson, Brendan J. O’Brien, Scott A. Nankervis, Matthew Denniff, Jack T. Harvey, Francine Z. Marques, Veryan Codd, Ewa Zukowska-Szczechowska, Nilesh J. Samani, Maciej Tomaszewski, Fadi J. Charchar

**Affiliations:** 1 School of Health Sciences, University of Ballarat, Mt Helen, Australia; 2 Department of Cardiovascular Sciences, University of Leicester and National Institute for Health Research (NIHR) Leicester Cardiovascular Biomedical Research Unit, Glenfield Hospital, Leicester, United Kingdom; 3 Department of Internal Medicine, Diabetology and Nephrology, Medical University of Silesia, Zabrze, Poland; University of Newcastle, United Kingdom

## Abstract

Telomere length is recognized as a marker of biological age, and shorter mean leukocyte telomere length is associated with increased risk of cardiovascular disease. It is unclear whether repeated exposure to ultra-endurance aerobic exercise is beneficial or detrimental in the long-term and whether it attenuates biological aging. We quantified 67 ultra-marathon runners’ and 56 apparently healthy males’ leukocyte telomere length (T/S ratio) using real-time quantitative PCR. The ultra-marathon runners had 11% longer telomeres (T/S ratio) than controls (ultra-marathon runners: T/S ratio = 3.5±0.68, controls: T/S ratio = 3.1±0.41; β = 0.40, SE = 0.10, *P = *1.4×10^−4^) in age-adjusted analysis. The difference remained statistically significant after adjustment for cardiovascular risk factors (*P = *2.2×10^−4^). The magnitude of this association translates into 16.2±0.26 years difference in biological age and approximately 324–648bp difference in leukocyte telomere length between ultra-marathon runners and healthy controls. Neither traditional cardiovascular risk factors nor markers of inflammation/adhesion molecules explained the difference in leukocyte telomere length between ultra-marathon runners and controls. Taken together these data suggest that regular engagement in ultra-endurance aerobic exercise attenuates cellular aging.

## Introduction

Regular high intense physical activity leads to an increase in cardio-respiratory fitness, which is thought to lead to subsequent reduction in risk of cardiovascular and total mortality [1], [2], [3]. Perplexingly, the anti-aging effect seems to be partly independent of traditional cardiovascular and metabolic risk factors [4], [5].

Telomeres are the repeated DNA sequence located at the distal ends of linear chromosomes [6]. Without the addition of telomeric repeats by the enzyme, telomerase, somatic cell telomeres progressively shorten with each round of cell division [7]. Therefore, telomere length is a well-known indicator of mitotic replicative history and biological age. Accumulating evidence suggests that moderate amounts of physical exercise correlates with longer leukocyte telomere length [8], [9], [10]. Although moderate exercise has been shown as beneficial in the prevention of cardiovascular disease, chronic, excessive sustained endurance exercise such as ultra-marathon running has been reported to cause nil or even adverse effects particularly for the heart and large arteries [11]. Association studies between endurance exercise and telomere length have shown conflicting results. Previous marathon runners were found to exhibit unchanged telomere lengths in differentiated granulocytes, lymphocytes and muscle cells compared to sedentary controls [12], [13]. In contrast, other studies have shown that endurance-trained athletes exhibit longer leukocyte telomeres [14], [15]. Therefore, the impact of repeated, ultra-endurance aerobic exercise on telomere length and biological aging remains unclear.

An ultra-marathon is an example of extreme exposure to ultra-endurance aerobic exercise – athletes run in excess of 42 kilometers in one day. The ultra-marathon runners are an excellent model of fitness induced by repeated engagement in ultra-endurance aerobic exercise. We have previously demonstrated ultra-marathon runners have exceptionally suppressed levels of low-grade inflammation and lower levels of low-density lipoprotein (LDL) cholesterol when compared to apparently healthy controls [16], [17]. However, it is not clear whether ultra-marathon runners also benefit from attenuation of biological aging, independent of reduction in measures of cardiovascular risk. Conversely, the exposure to ultra-endurance aerobic exercise was associated with increased oxidative stress [18] that is a well-known correlate of telomere attrition rate in cellular studies [19].

Here, we measured leukocyte telomere length of male ultra-marathon runners who regularly engage in ultra-endurance running and compared them to apparently healthy controls from the general population. We also investigated whether traditional cardiovascular risk factors (including blood pressure – BP – and lipids) as well as adhesive molecules and markers of inflammation play a role in the association between ultra-endurance aerobic exercise and telomere length.

## Materials and Methods

### Clinical Phenotyping

Sixty-seven male ultra-marathon runners and 63 age and sex-matched apparently healthy controls were included in this study. All individuals were of the same ethnicity (white Polish). The participants demographics have been outlined previously [17]. Briefly, the ultra-marathon runners had completed at least two ultra-marathons, had an average training distance of 40–100km per week and had trained for a minimum of two years [17]. All participants gave informed written consent and the study was approved by the University of Ballarat Human Research Ethics Committee. (Methods S1).

### Biochemical Analysis

The biochemical analyses were described before [16], [17] (Methods S1).

### Telomere Length Quantification

DNA from was extracted in the same laboratory from peripheral whole-blood by methods described elsewhere [17]. Telomere length was measured using an established quantitative real-time PCR technique [20]. This method expresses telomere length as a ratio (T/S) of telomere repeat length (T) to copy number of a single copy gene, 36B4 (S), within each sample (Methods S1).

### Statistical Analysis

Phenotypes with non-normal distribution underwent log-transformation before further analysis. The Student’s t-test or Mann-Whitney U-test were used to examine crude differences in quantitative traits between the two groups. Linear correlation estimates were calculated using Pearson’s method. Linear regression models were used to analyze telomere length in ultra-marathon runners and controls using multiple regression analyses with adjustment for age and other phenotypic covariates, and stepwise selection following adjustment for covariates. Significance was determined as *P*<0.05. The difference in T/S ratio between ultra-marathon runners and controls was divided by the unstandardized β-coefficient from linear regression model including age and T/S ratio from a large population-based study (n >45,000), conducted at the same laboratory using identical methodologies [21]. This provides an estimate of difference in biological age between ultra-marathon runners and controls using age-related telomere attrition rate. Others who have quantified telomere length by measuring terminal restriction fragments using the Southern Blot technique, have shown the age-related telomere attritions is approximately 20–40 base pairs (bp) per year [21]. To estimate the bp telomere length difference between cohorts, we multiplied the biological age difference (16.2 years) between ultra-marathon runners and controls by the average bp decline per year previously described –20–40bp [21]. In doing so, we were able to estimate the approximate difference in telomere length (expressed as bps) between ultra-marathon runners and controls.

## Results

### Subject Demographics

The demographic and phenotypic data are displayed in Table 1.

**Table 1:** Clinical phenotypes of ultra-marathon runners and apparently healthy controls.

Ultra-marathonrunners (n = 67)		Controls (n = 56)	*P*-value
Age (years)	43.6±9.2	42.8±9.2	0.62
BMI (kg/m^2^)	23.2±2.0	25.2±2.5	**2.7**×**10** ^−**6**^
MAP	95.8±5.4	95.9±8.2	0.92
TC (mmol/L)	5.1±1.0	5.7±1.0	**0.0014**
HDL-C (mmol/L)	1.2±0.3	1.0±0.3	**6.7**×**10** ^−**4**^
Triglycerides(mmol/L)*	1.7 (1.37, 1.86)	1.6 (1.37, 1.86)	0.70
CRP (mg/L)*	0.4 (0.34, 0.59)	1.4 (1.05, 1.91)	**2.6**×**10** ^−**8**^
IL-6 (pg/mL)*	1.3(1.11, 1.46)	1.5 (1.26, 1.74)	0.10
Leptin (ng/mL)*	2.1 (1.76, 2.40)	5.6 (4.34, 7.20)	**3.6**×**10** ^−**9**^
sE-selectin (ng/mL)*	49.8 (44.51, 55.76)	46.1 (40.67, 52.18)	0.46
sICAM -1 (ng/mL)*	202.1 (186.53, 219.06)	232.8 (210.38, 257.56)	**0.015**

Data are from either Student’s t-test or Mann-Whitney U-tests and are expressed as means and standard deviations or geometric means and 95% confidence intervals (*); BMI – body mass index, MAP – mean arterial pressure, TC – total cholesterol, HDL – high-density lipoprotein cholesterol, CRP – C-reactive protein, IL-6– interleukin-6, sICAM-1– soluble intercellular.

Ultra-marathon runners had significantly lower mean body mass index (BMI), total cholesterol (TC), soluble intracellular adhesion molecule (sICAM-1), leptin and C-reactive protein (CRP), and significantly higher mean high density lipoprotein (HDL)-cholesterol than controls.

### Telomere Length and Aging

**Figure 1:**
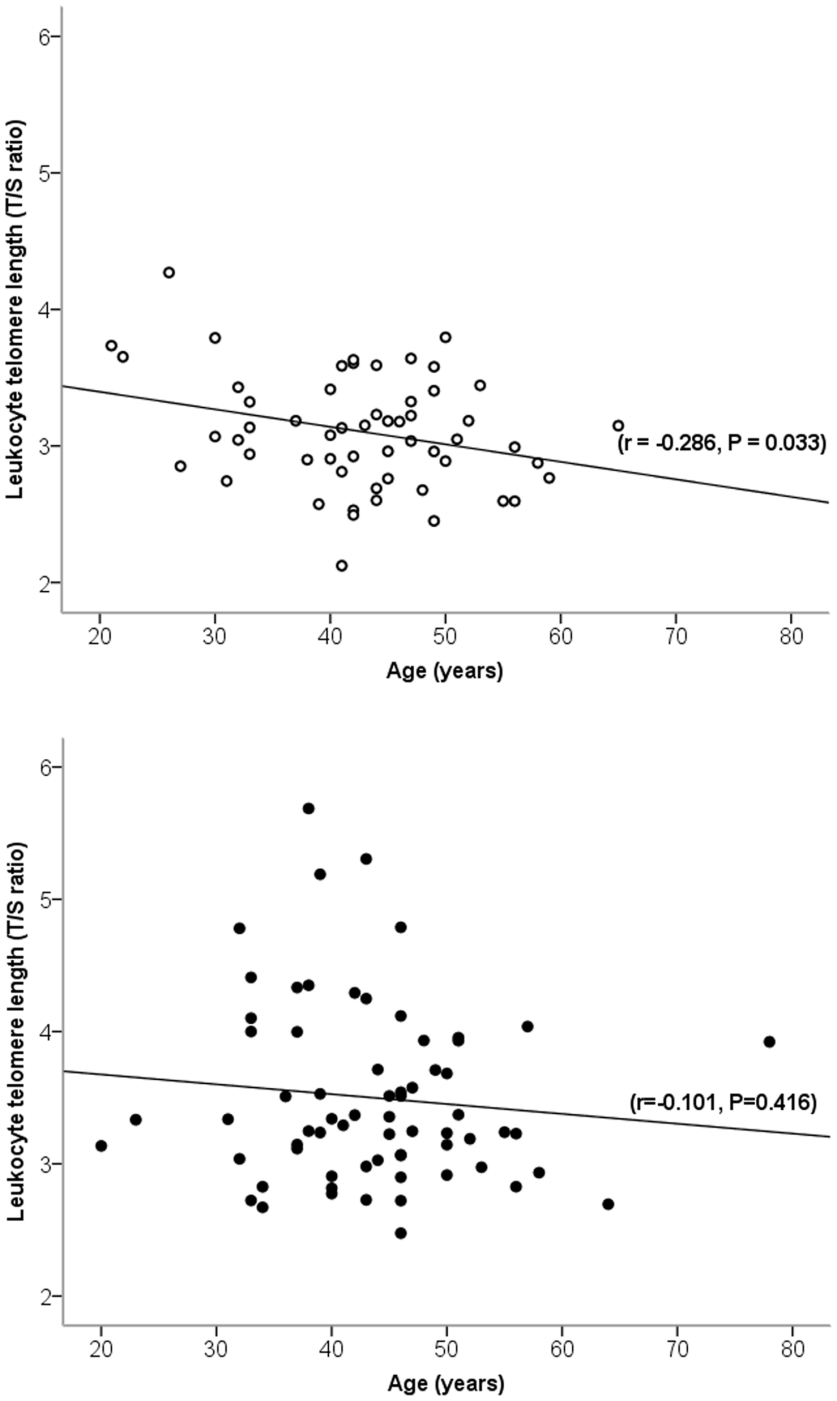
Pearson's linear correlation between age and leukocyte telomere length in ultra-marathon runners and controls. Ultra-marathon runners are indicated by filled circles and controls are indicated by empty circles.

**Figure 2:**
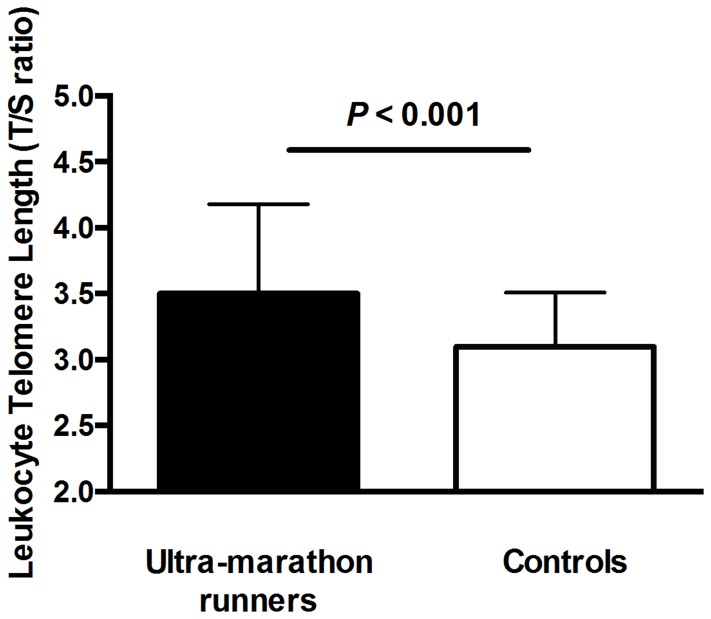
Telomere length comparison between ultra-marathon runners and controls. Mean leukocyte telomere length is presented in arbitrary units as the telomere to single copy gene (T/S) ratio. Error bars represent standard deviation.

Age was inversely related to telomere length in controls (r = −0.29) and weakly – in ultra-marathon runners (r = −0.10) (Figure 1). The rate of telomere attrition (slopes of negative correlation between age and telomere length), however, was not statistically different between cohorts (*P = *0.64) (Figure 1). The ultra-marathon runners had an 11% longer telomere length (T/S ratio) than controls (ultra-marathon runners: 3.5±0.68, controls: 3.1±0.41; β = 0.40, SE = 0.10, *P = *1.4×10^−4^) in age-adjusted analysis (Figure 2).

The difference remained statistically significant after adjustment for differences between ultra-marathon runners and controls (age, BMI, TC, HDL-C, CRP, leptin, sICAM-1, PCR Plate, β = 0.44, SE = 0.14, *P = *2.2×10^−4^) (Table 2). In the stepwise regression model (adjusting for age, interleukin-6– IL-6, mean arterial pressure – MAP and PCR Plate) the telomere length was significantly higher in ultra-marathon runners than controls (β = 0.44, SE = 0.10, *P* = 4.2×10^−5^) (Table 2). After full adjustment, we estimated that the difference in biological aging between ultra-marathon runners and controls was approximately 16.2±0.26 years. Therefore, we estimate the ultra-marathon runners have on average approximately 324–648bp longer leukocyte telomeres compared to those of their less active peers.

**Table 2:** Difference in leukocyte telomere length between ultra-marathon runners and controls.

Model	Covariates adjusted for	β-coefficient (95%CI)	*P*-value
Basic	Age	0.40 (0.103)	**1.4×10** ^−**4**^
Fully adjusted model 1 (stepwise)	Age, IL-6, MAP and PCR Plate	0.44 (0.103)	**4.2×10** ^−**5**^
Fully adjusted model 2 (forced)	Age, BMI, TC, HDL-C, CRP, leptin, sICAM-1, PCR Plate	0.44 (0.140)	**2.2**×**10** ^−**4**^

The differences are expressed as unstandardized β-coefficients with standard errors from either stepwise linear regression (Fully adjusted model 1) or linear regression (Fully adjusted model 2– Forced), MAP – mean arterial pressure, IL-6– interleukin-6, PCR Plate – experiment used in measurement in LTL, BMI – body mass index, TC – total cholesterol, HDL-C – high-density lipoprotein cholesterol, CRP – C-reactive protein, sICAM – soluble intercellular adhesion molecule-1.

### Telomere Length and Cardiovascular Risk Factors

Apart from MAP, conventional cardiovascular risk factors (BMI, TC, HDL-cholesterol and triglycerides) were not associated with telomere length in ultra-marathon runners and controls (Table 3).

**Table 3:** Linear correlation between leukocyte telomere length and cardiovascular health markers, adhesion molecules, cytokines and inflammation markers.

	All			Ultra-marathon runners			Controls			
	r	*P*-value		r	*P*-value		r		*P*-value	
**Cardiovascular risk factors**										
BMI (kg/m^2^)	−0.13		0.15	0.08		0.50		−0.13		0.33
TC (mmol/L)	−0.04		0.69	0.05		0.69		0.14		0.33
HDL-C (mmol/L)	0.03		0.76	−0.11		0.37		−0.04		0.75
MAP	0.13		0.16	**0.30**		**0.015**		−0.03		0.80
Triglycerides (mmol/L)	0.02		0.85	0.10		0.42		0.13		0.35
**Inflammation/adhesion molecules**										
CRP (mg/L)	−0.09		0.29	0.11		0.37		−0.03		0.84
IL-6 (pg/mL)	−0.12		0.20	−0.10		0.44		−0.06		0.68
Leptin (ng/mL)	−0.15		0.09	0.006		0.96		0.07		0.62
sE-selectin (ng/mL)	0.02		0.85	0.01		0.92		−0.06		0.67
sICAM -1 (ng/mL)	−0.14		0.11	−0.05		0.69		−0.16		0.25

BMI – body mass index, TC – total cholesterol, HDL-C – high-density lipoprotein cholesterol, MAP – mean arterial pressure, CRP – C-reactive protein, IL-6– interleukin-6, sE-selectin – serum E-selecin, sICAM-1– Soluble intercellular adhesion molecule-1. Data from Pearson’s Correlations are expressed by r and p-values.

There was a positive correlation between MAP and telomere length in ultra-marathon runners (r = 0.30, *P = *0.015). CRP, leptin, adhesion molecules (serum E-selectin – sE-selecting – and sICAM-1) and IL-6, were not significantly associated with telomere length in ultra-marathon runners, controls or joint analysis of both groups (Table 3).

## Discussion

To our knowledge, this is the largest study to show that ultra-marathon runners exhibit markedly longer leukocyte telomere length compared to age-matched apparently healthy controls who do not engage in ultra-endurance aerobic exercise. We also show the impact of aging on telomere length is attenuated in ultra-marathon runners and that telomeres are approximately 16.2 years biologically younger compared to less active controls. Our results support previous data obtained from endurance-trained athletes (engaging in a similar volume of aerobic exercise) and sedentary controls [14]. However, we show that the difference in telomere length between ultra-marathon runners and controls cannot be simply explained by better cardiovascular risk profile.

Investigations on the effect of aerobic exercise on telomere length has so far provided no conclusive information on how much exercise is optimal and safe for immune cell chromosomal stability [8], [9], [10], [12], [14], [22]. Sedentary middle-aged individuals exhibit shorter telomere length compared to younger and age-matched track and field athletes and endurance-trained athletes (marathon runners and triathletes) [14]. The analysis of telomere length in twin volunteers revealed the more physically active twin had longer telomeres than the less active twin [8]. Furthermore, exercise intensity is beneficial for telomere dynamics in women, as telomere length was positively associated with engaging in more frequent vigorous physical activity [23] and vigorous physical activity ameliorated telomere attrition caused by psychological stress [24]. Telomere length was also positively correlated with maximal oxygen uptake in older (55–72years) participants and it was suggested that telomere erosion was attenuated in middle-aged participants who exercise regularly [25]. In contrast, daily amount of energy expenditure had an inverted ‘U’-type relationship with telomere length, in that moderate (991–2340 Kcal^.^wk^−1^) levels of energy expenditure were associated with longer telomeres compared to very low (<991 Kcal^.^wk^−1^) and high energy expenditures (>3541 Kcal^.^wk^−1^) [9]. We have clearly demonstrated that men who engage in ultra-endurance aerobic exercise have significantly longer telomeres compared to those who did not exercise extensively on a regular basis but otherwise were apparently healthy. Recently, marathon runners were reported to have similar lymphocyte and granulocyte telomere lengths compared to controls [12]. Potential explanations for the discrepancy between the previous findings [12] and our results may be due to the larger sample size of our study, greater age-range of participants and also due to the higher volume of aerobic exercise performed by the ultra-marathon runners included in our investigation. Interestingly, skeletal muscle telomeres are longer in endurance-trained cross-country skiers’ compared to non-athletes [26]. Given the synchrony between leukocyte and skeletal muscle cell telomere shortening [27], our data along with others’ [14], [15], [26] support the hypothesis that both endurance and ultra-endurance exercise are beneficial to leukocyte telomere maintenance.

The longer telomeres observed in the ultra-marathon runners in our study may be a result of increased telomerase expression in leukocytes as a previous study by Werner et al. [14] showed that young and middle-aged athletes, had increased telomerase activity compared to sedentary controls [14]. Werner et al. [14] also found that athletes have differentially expressed genes associated with the shelterin complex (*TRF2*, *CHK2*, Ku 70 and 80) compared to sedentary controls [14]. Moreover, a significant increase in telomerase activity in mononuclear cells was observed after a three month intervention including 30 minutes of moderate physical activity, six days a week [28]. Recently, it was reported that following a seven day ultra-marathon footrace, ultra-marathon runners exhibited greater leukocyte mRNA content of shelterin associated genes – *TRF1, TRF2* and *POT1* [29]. The above proteins, along with several others, protect chromosomal and telomere integrity through the formation of the shelterin complex [30]. Therefore, endurance-trained individuals may benefit from ameliorated leukocyte telomere attrition by modulated shelterin and telomerase dynamics.

Our data also suggest that the difference in telomere length between ultra-marathon runners and controls cannot be simply explained by better cardiovascular risk profile in those who engage in regular ultra-endurance aerobic exercise. Indeed, neither traditional cardiovascular risk factors nor markers of inflammation/adhesion molecules showed association with telomere length, and their inclusion in the regression model had no effect on the association between telomere length and ultra-endurance aerobic exercise. Although there was no significant difference in the MAP between the ultra-marathon runners and controls we observed a positive correlation between leukocyte telomere length and MAP in ultra-marathon runners but not the controls. The biological mechanisms of this somewhat paradoxical correlation are not clear. Interestingly, previous findings have shown that telomere length is positively related to left ventricular mass [31], that in turn is a direct associate of blood pressure. In this context the correlation seen in our study may be explained (at least in part) by the adaptation to chronic endurance exercise. On the other hand, we should acknowledge that blood pressure is a rapidly changing physiological parameter and the value of single clinic measurements may not necessarily reflect the long-term effect of BP on cardiovascular system, in particular when taken in a relatively small group of individuals. Larger population samples are necessary to fully dissect the association between BP and telomere length in ultra-marathon runners.

We should, however, acknowledge that several unmeasured intermediate phenotypes may be relevant here. Although not measured directly, cardiorespiratory fitness gained from previous extensive training would be significantly better in the ultra-marathon runners than controls.

Maximal oxygen uptake has been positively correlated with telomere length in older, endurance-trained adults [25]. Interestingly, patients with longer telomeres and greater exercise capacity had reduced mortality risk [32]. Therefore, it is tempting to postulate that increasing amounts of ultra-endurance aerobic exercise may be beneficial to decreasing mortality risk through cardiovascular training adaptations, potentially leading to an extended lifespan.

In the current study we found that biologically ultra-marathon runners are approximately 16.2 years younger than less physically active controls, equating to an approximate 324–648bp longer telomeres than controls. Notably, endurance-trained athletes’ (>55years) telomeres, measured by Southern Blot, were shown to have approximately 900bps longer leukocyte telomeres than sedentary individuals [15]. Engaging in greater amounts of physical activity has been shown previously to have anti-aging effects. Ultra-endurance athletes have 17% greater longevity compared to the general population [33], and numerous studies have demonstrated decreased mortality with more frequent exercise [3], [34]. With telomere length a marker of biological age, less active individuals exhibit 10 years biologically older leukocytes compared to their more active peers [8]. Healthy individuals have 11 years biologically younger leukocytes compared to patients with CVD [35]. Moreover, coronary artery disease patients with greater exercise capacity exhibited longer telomeres compared to patients with a lower exercise capacity, representing a four year difference in biological age [32]. In this context, a 16 year difference in biological age between ultra-marathon runners and controls appears particularly significant and its implications for health and disease needs to be further elucidated.

Our study has a number of limitations. Information on diet [36] and psychological stress [37] which have been demonstrated to influence telomere dynamics were not recorded in our participants. Our study was cross-sectional in nature and therefore we were unable to assign direct causative nature to the association between telomere attrition and physical exercise. Future studies should investigate telomere erosion longitudinally, measuring telomeres at multiple time points in people engaging in different physical activity levels, to gain a better insight into the protective effect physical exercise may have on cellular aging. Moreover, delineation of the molecular pathways modulated by exercise, which are responsible for telomere maintenance, is of high priority.

In conclusion, our results are the first to demonstrate that chronic ultra-endurance aerobic exercise is associated with slower cellular aging by attenuated telomere length attrition, independent of age and traditional markers of cardiovascular risk, as well as markers of inflammation/adhesion molecules. They also demonstrate that ultra-endurance exercise does not have adverse effects on the cardiovascular system through telomere attrition.

## Supporting Information

Methods S1 **Supplementary Methods.**
(DOCX)
