# Supplementary material for: Longer Leukocyte Telomeres Are Associated with Ultra-Endurance Exercise Independent of Cardiovascular Risk Factors
Source: PLoS One. 2013 Jul 31;8(7):e69377. doi: 10.1371/journal.pone.0069377 (PMC3729964; doi:10.1371/journal.pone.0069377)
Supplement: Methods S1 — Supplementary Methods. (DOCX) [file pone.0069377.s001.docx]

**Methods S1**

All ultra-marathon runners were recruited prior to the 100km Calissia 2000 foot-race. All controls were apparently healthy, were not taking any medications and were not engaging in regular intense physical exercise. Of the 63 controls recruited into this study, 56 had DNA available for telomere measurements. All participants gave informed written consent and the study was approved by the University of Ballarat Human Research Ethics Committee.

Phenotyping included clinical history (using a standardized questionnaire), physical examination, height and weight as well as resting BP measurements, along with a fasting blood sample obtained for biochemical analysis as previously described one day prior to the ultra-marathon event [[1](#_ENREF_1)].

Briefly, circulating concentrations of TC, HDL-C and triglycerides were measured using enzymatic methods. sICAM-1 and sE-selectin concentrations were assessed by quantitative sandwich enzyme immunoassay technique (R&D Systems) [[1](#_ENREF_1)]. Serum CRP was quantified using a sensitive double antibody sandwich ELISA with rabbit antihuman CRP and peroxidise-conjugate rabbit anti-human CRP, defined previously [[2](#_ENREF_2)]. Serum leptin concentration was quantified using an in-house radioimmunoassay outlined by others [[3](#_ENREF_3)]. IL-6 was measured using a high-sensitive human IL-6 immunoassay (R&D Systems).

Amplification reactions were set up using a Qiagiligy liquid handling robot (Qiagen, UK) and performed in a Qiagen Rotor-Gene Q (Qiagen, UK). Real-time PCR was performed in duplicates with sensiMix NoRef SYBR Green master mix (Bioline, UK), primer sets as previously described [[4](#_ENREF_4)] and 30ng of DNA. Reactions were prepared to a final volume of 25µl and both no-template and positive (genomic DNA from the K562 cell line) control duplicates were included in all experiments. The cycling conditions used were as previously described [[4](#_ENREF_4)]. In order to standardize across PCR runs the T/S ratio was calculated relative to a calibrator sample (genomic DNA from the K562 (ATCC) cell line) included on each plate, using the Qiagen comparative quantification software as previously described [[4](#_ENREF_4)]. As a quality control, one plate containing randomly selected duplicates of the same 48 DNA samples (used in the previous experiments), no template controls and calibrators (K562) was assayed a second time to assess the reproducibility of the data. The inter-assay coefficient of variation for the T/S ratio was 2.6% and the correlation (r) between the two runs was 0.98, demonstrating the integrity of the assay and acceptable reproducibility. The intra-assay coefficient of variation expressed as a percentage was 1.7% for the telomere primer set and 1.6% for the 36B4, respectively.

1. Tomaszewski M, Charchar FJ, Przybycin M, Crawford L, Wallace AM, et al. (2003) Strikingly low circulating CRP concentrations in ultramarathon runners independent of markers of adiposity: how low can you go? Arterioscler Thromb Vasc Biol 23: 1640-1644.

2. Freeman DJ, Norrie J, Caslake MJ, Gaw A, Ford I, et al. (2002) C-reactive protein is an independent predictor of risk for the development of diabetes in the West of Scotland Coronary Prevention Study. Diabetes 51: 1596-1600.

3. Wallace AM, McMahon AD, Packard CJ, Kelly A, Shepherd J, et al. (2001) Plasma leptin and the risk of cardiovascular disease in the west of Scotland coronary prevention study (WOSCOPS). Circulation 104: 3052-3056.

4. Codd V, Mangino M, van der Harst P, Braund PS, Kaiser M, et al. (2010) Common variants near TERC are associated with mean telomere length. Nat Genet 42: 197-199.
